# COVID-19 Vaccines, Effectiveness, and Immune Responses

**DOI:** 10.3390/ijms232315415

**Published:** 2022-12-06

**Authors:** Haneen Imad Abufares, Leen Oyoun Alsoud, Mohammad A. Y. Alqudah, Mohd Shara, Nelson C. Soares, Karem H. Alzoubi, Waseem El-Huneidi, Yasser Bustanji, Sameh S. M. Soliman, Mohammad H. Semreen

**Affiliations:** 1 College of Pharmacy, University of Sharjah, Sharjah P.O. Box 27272, United Arab Emirates; 2 Sharjah Institute for Medical Research, University of Sharjah, Sharjah P.O. Box 27272, United Arab Emirates; 3 College of Medicine, University of Sharjah, Sharjah P.O. Box 27272, United Arab Emirates

**Keywords:** SARS-CoV-2, COVID-19, vaccines, effectiveness, immune responses

## Abstract

The COVID-19 pandemic, caused by the severe acute respiratory syndrome coronavirus 2 (SARS-CoV-2), has captivated the globe’s attention since its emergence in 2019. This highly infectious, spreadable, and dangerous pathogen has caused health, social, and economic crises. Therefore, a worldwide collaborative effort was made to find an efficient strategy to overcome and develop vaccines. The new vaccines provide an effective immune response that safeguards the community from the virus’ severity. WHO has approved nine vaccines for emergency use based on safety and efficacy data collected from various conducted clinical trials. Herein, we review the safety and effectiveness of the WHO-approved COVID-19 vaccines and associated immune responses, and their impact on improving the public’s health. Several immunological studies have demonstrated that vaccination dramatically enhances the immune response and reduces the likelihood of future infections in previously infected individuals. However, the type of vaccination and individual health status can significantly affect immune responses. Exposure of healthy individuals to adenovirus vectors or mRNA vaccines causes the early production of antibodies from B and T cells. On the other hand, unhealthy individuals were more likely to experience harmful events due to relapses in their existing conditions. Taken together, aligning with the proper vaccination to a patient’s case can result in better outcomes.

## 1. Introduction

Coronaviruses are well-known zoonotic enveloped single-stranded ribonucleic acid viruses [1]. Ribonucleotide viruses have a high mutation capability, which explains their progressive transmissibility and infectivity [2]. This viral infection can range from mild asymptomatic to severe, which leads to hospitalization due to respiratory distress, including chest pain, shortness of breath accompanied by low blood oxygen, and loss of motor functions [1]. The human coronavirus-related illness emerged in November 2002 when patients developed atypical pneumonia caused by acute respiratory syndrome coronavirus (SARS-CoV-1) infection [3]. In 2012, a fatal, widely spreading coronavirus strain called the Middle East respiratory syndrome (MERS) emerged in the Middle East. Patients with MERS presented with acute respiratory and renal failure, severe complications in different organs, tissues, and body fluids, and pericardium inflammation and coagulation [4]. In December 2019, a new coronavirus, SARS-CoV-2, was reported in Wuhan city, China, with an unusual number of patients experiencing severe acute respiratory pneumonia [1]. The 14 open reading frames (ORFs) in the SARS-CoV genome code for 27 proteins. Nearly 70% of the virus genome is made up of the first ORF (ORF1a and ORF1b), which include 15 non-structural proteins (nsps). The four major structural proteins are membrane (M), envelope (E), spike (S), and nucleocapsid (N) proteins, which were encoded by the remaining ORFs in the 3’-terminal region, together with eight accessory proteins (3a, 3b, p6, 7a, 7b, 8b, 9b, and ORF14) [5]. Moreover, comparing SARS-CoV-2 to SARS-CoV at the amino acid level indicated that both viruses are relatively similar [5]. However, SARS-CoV-2 is distinct from MERS-CoVs. Phylogenetic analysis based on whole-genome sequence revealed that SARS-CoV-2 has descended from the SARS-like bat CoV lineage [6,7].

With the extraordinary increase in the number of cases and deaths worldwide, the World Health Organization (WHO) declared that SARS-CoV-2 is a global pandemic and named the disease Coronavirus Disease 2019 (COVID-19) in March 2020 [8]. Globally, as of 5 August 2022, there were 578,142,444 million confirmed COVID-19 cases, with more than 6.4 million deaths [9]. A massive economic burden has been imposed on patients and the general population due to the COVID-19 pandemic [10]. Therefore, the need for effective medications and vaccines against COVID-19 was urgent. Consequently, several pharmaceutical companies compete to develop vaccines that can minimize the damage associated with COVID-19. 

As a result, the Pfizer/BioNTech vaccine was added to the WHOs emergency use list on 31 December 2020, followed by AstraZeneca/Oxford, Janssen, Moderna, Sinopharm, Sinovac-CoronaVac, Covaxin, Covovax, and Convidecia COVID-19 vaccines. These vaccines have met the necessary criteria for safety and efficacy, according to the WHO [11]. It has been demonstrated that the S glycoprotein of SARS-CoV-2 is the optimal target for vaccine development on various platforms due to its high antigenicity and capacity to elicit potent immune responses [12]. Furthermore, the epitopes derived from S glycoprotein utilize peptide fragments to minimize the allergic and/or reactogenic effects, offering a promising alternative for creating new and safer vaccinations with highly targeted immune responses [13]. Vaccines based on the S protein have the potential to induce antibodies that either neutralize virus infection or prevent virus binding and fusion [14].

This review discusses the WHO-approved COVID-19 vaccine types, efficacy, safety concerns, complications, and related immune responses.

### 1.1. SARS-CoV-2 Vaccines 

**Figure 1:**
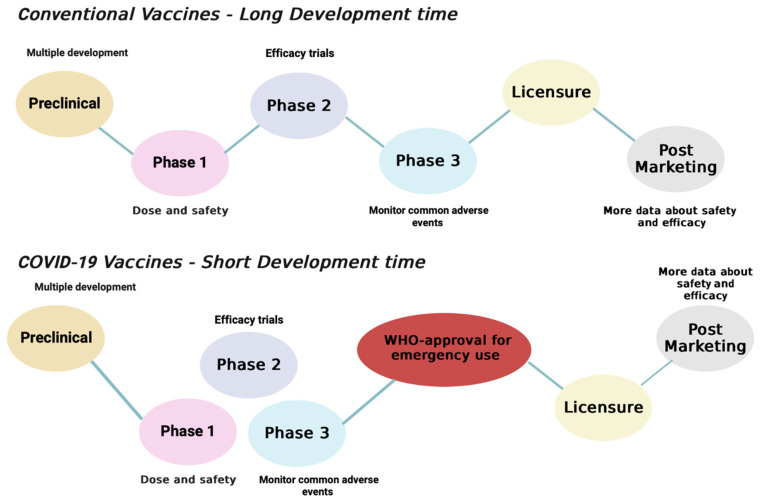
Development of COVID-19 vaccine in comparison to other vaccines.

The COVID-19 vaccine has been developed in a remarkable time due to the pandemic. Phase I was completed in 6–9 months, compared to the normal time frame of 3–9 years for other vaccines (Figure 1) [15]. In addition, the three phases of COVID-19 vaccine development overlapped to speed up the process [16].

There are four primary categories of COVID-19 vaccines, including (i) attenuated whole virus vaccines, (ii) protein-based vaccines, (iii) viral vector vaccines, and (iv) nucleic acid vaccines.

### 1.2. Whole Virus Vaccines

**Figure 2:**
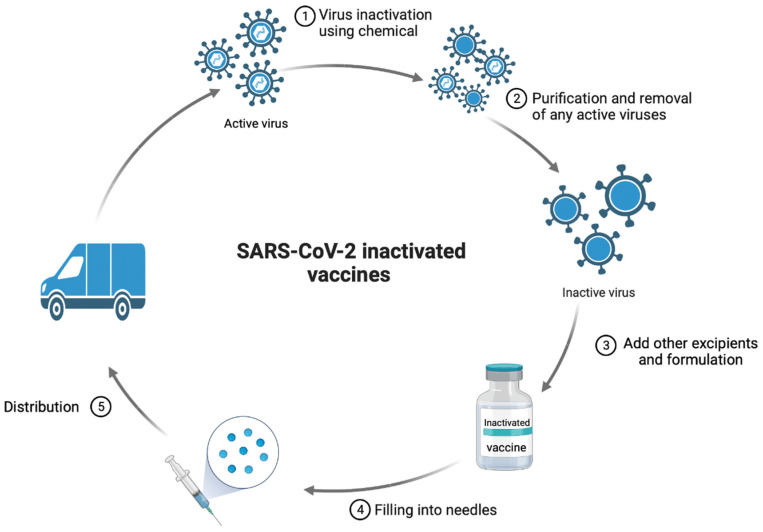
COVID-19 whole virus vaccine development.

Whole virus vaccines are attenuated or inactivated viral vaccines, which are developed by destroying the virus’ genetic material by heating, chemicals, or radiation while maintaining all viral proteins intact (Figure 2). Therefore, the whole virus vaccines are not infective but can still stimulate the immune system [17]. Whole inactivated virus vaccines can produce a robust immune response. Antigens from SARS-CoV-2 will be presented to antigen-presenting cells that produce immune responses and memory cells [18]. Approved COVID-19 whole virus vaccines include Sinopharm, Sinovac, and Covaxin. Table 1 illustrates the effectiveness, pros, cons, and usage of whole virus COVID-19 vaccines.

**Table 1:** WHO-approved vaccines.

WHO-Approved Inactivated Whole SARS-CoV-2 Vaccines					
The Vaccine	Efficacy	Advantages	Disadvantages	Usage	References
Sinopharm	78.10% in preventing symptomatic COVID-19 infection.	Easy to preserve, manufacture, and transport.	Reduction in the efficacy of vaccines over time can lead to lower immunogenicity.	Sinopharm inactivated vaccines are given in 2 doses for 18 years and older individuals.	[19]
Sinovac	51% in preventing symptomatic COVID-19 infection.	Easy to preserve, manufacture, and transport.	The need of batch control to prevent any impairment that can lead to infection.	Sinovac inactivated vaccines are given in 2 doses for 18 years and older individuals.	[19]
Covaxin	77.80% in preventing symptomatic COVID-19 infection.	Easy to preserve, manufacture, and transport.	The need of adjuvant to boost the immunity.	Covaxin inactivated vaccines are given in 2 doses for 18 years and older individuals.	[20]
Nucleic acid based WHO-approved COVID-19 vaccines					
The vaccine	Efficacy	Advantages	Disadvantages	Usage	References
Pfizer/BioNTech	91.10% in reducing symptomatic COVID-19	Safe for children along with long-term protection against COVID-19 infection. High immunogenicity	The need of ultra-low temperature for transportation and storage. They are expensive	Vaccines are given in 2 doses for 6 months and older individuals, with dose modification in younger patients.	[21,22]
Moderna	94.1% in reducing symptomatic COVID-19	High immunogenicity	The need of low temperature for transportation and storage.	Vaccines are given in 2 doses for 6 months and older individuals, with dose modification in younger patients	[23,24]
Viral vector WHO-approved COVID-19 vaccines					
The vaccine	Efficacy	Advantages	Disadvantages	Usage	References
AstraZeneca	79% in preventing symptomatic COVID-19 infection	Stable and easier to distribute	The need for adjuvant and the limited immunogenicity	AstraZeneca is given in 2 doses for 18 years and older individuals.	[25,26]
Johnson and Johnson	67% in preventing symptomatic COVID-19 infection	Stable and easier to distribute as well as it is given in a single shot	The need for adjuvant and the limited immunogenicity	Johnson and Johnson vaccine is given in 1 dose for 18 years and older individuals.	[27]

### 1.3. Protein-Based Vaccines

**Figure 3:**
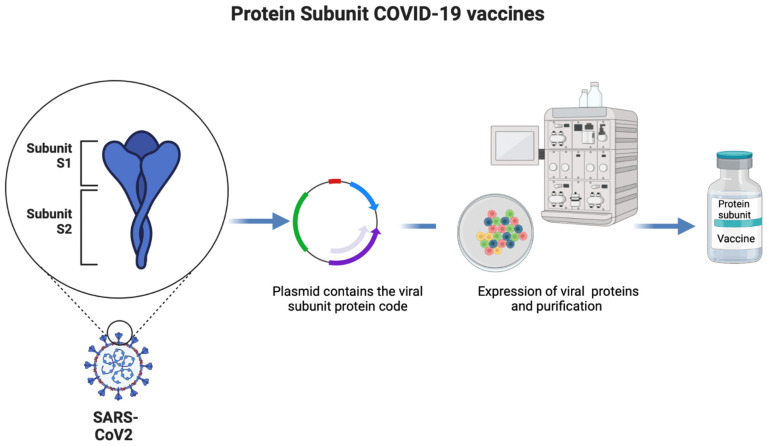
Development of COVID-19 protein subunit vaccine.

Protein-based vaccines use only a part of the viral protein as an antigen to develop an effective host immune response [28]. They can be classified into subunit and virus-like particle vaccines. The subunit vaccines are prepared using recombinant technology (Figure 3). This technology is relatively expensive compared to other techniques, but it is safe, efficient, and accessible [28]. The main disadvantage of the recombinant protein vaccine is its low immunogenicity and the need for using an adjuvant. An example of SARS-CoV-2 protein-based vaccines includes Covovax (Novavax) [28]. The main advantage of Novavax is its unique platform through inserting part of the spike protein that will produce a more balanced and sustained immune response compared to other vaccines, along with the ability to withstand the external environment [29]. On the other hand, the main disadvantages are the need for longer research time and effort to develop the protein subunit vaccine and the need to use an adjuvant to have a better immune response outcome. The Novavax vaccine is given in two doses for 18 years and above and has 89.7% efficacy in preventing symptomatic infection [29].

### 1.4. Nucleic Acid-Based Vaccines

**Figure 4:**
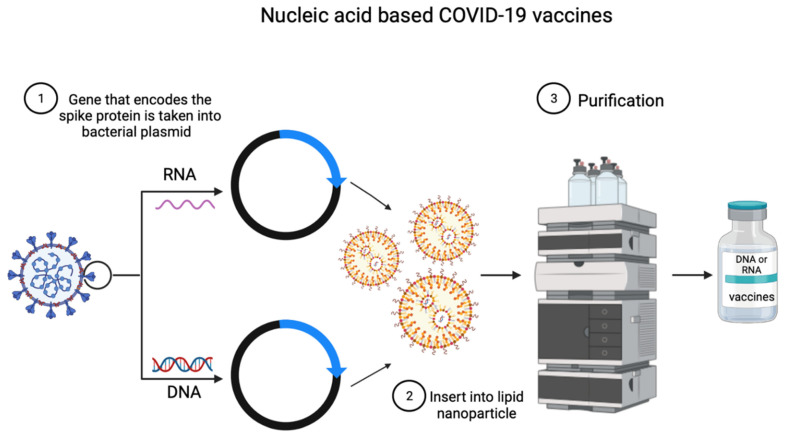
Development of COVID-19 nucleic acid-based vaccine.

The use of deoxyribonucleic acid (DNA) and ribonucleic acid (RNA) is a newly utilized technique for COVID-19 vaccine development [30]. They are developed recombinantly by using a specific virus gene, which is expressed in the body as a protein that causes antibody production (Figure 4) [30].

DNA and RNA vaccines can integrate into the human genome and mutate humans’ genetic code. However, RNA is very unstable compared to DNA, and the only RNA-approved vaccines for COVID-19 are Pfizer/BioNTech and Moderna [30,31]. Table 1 shows the efficacy, benefits, drawbacks, and application of the various nucleic acid COVID-19 vaccines.

### 1.5. Viral Vector-Based Vaccines

**Figure 5:**
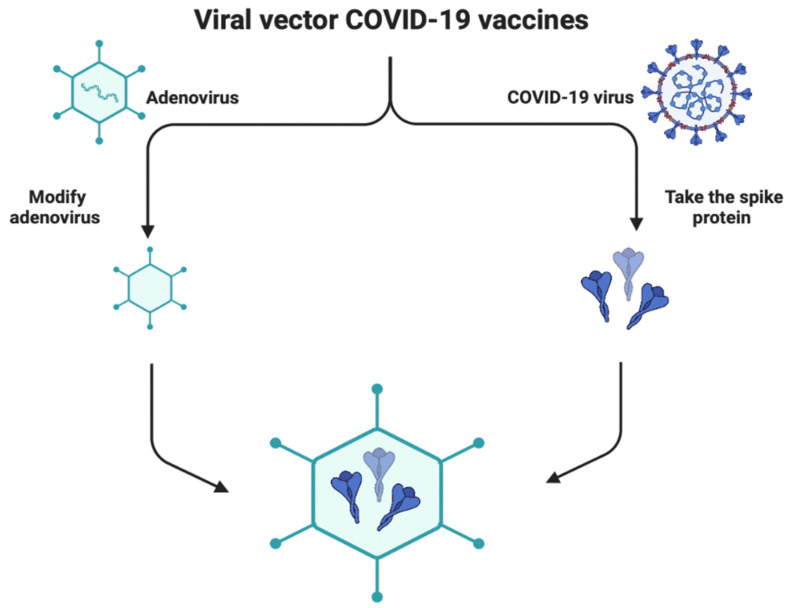
Development of COVID-19 viral vector vaccine.

The virus-like particles technology was used to develop different SARS-CoV-2 vaccines, such as AstraZeneca and Johnson & Johnson, by using a harmless virus as a vector to carry a SARS-CoV-2 antigen code (Figure 5). This technology produces a specific protein with the capability to build the host’s immune response [32]. The main disadvantage of viral vector vaccines is the need for multiple booster doses [32,33]. Table 1 shows the efficacy, strengths, weaknesses, and usage of various viral vector COVID-19 vaccines. Taken together, different vaccine platforms are essential for immunity to prevent the spread of COVID-19.

## 2. Vaccine Formulation and Their Complication 

Vaccine design is one of the crucial steps in developing an optimum immune response [34]. It depends on the antigen and platform selection, the need for adjuvants, the formulation, and the route of administration [35]. In the case of COVID-19 vaccines, the SARS-CoV-2 structure contains different proteins (S1 and S2 spike proteins), nucleocapsid protein, membrane protein, and envelope protein. S protein was used in most vaccines developed against SARS-CoV-2 [36]. 

To enhance the immune response, the vaccine formulation may require adjuvants [37]. The most used adjuvant in COVID-19 vaccines is aluminum salt, which includes either aluminum hydroxide or aluminum phosphate [38]. In addition to enhancing IgG1 titers and neutralizing antibodies, the adjuvant is durable and plays an essential role in reducing vaccine doses [39]. However, many previous studies showed that using small amounts of aluminum in the formulation of COVID-19 vaccines is considered safe [40]. Other studies reported that it could cause mild side effects such as allergies and pain at the injection site and, to a lesser extent, severe side effects including neurotoxicity, autism, and some chronic diseases [40,41,42].

Oil in water emulsion is another type of adjuvant used in the formulation of COVID-19 vaccines, including MF59 and AS03 [43]. This approach elicits a more balanced immune response, possibly by improving antigen uptake, attracting immune cells, and promoting antigen-presenting cell migration [43]. However, emulsion adjuvants can cause side effects ranging from mild headache, fever, and nausea to more severe, such as the induction of autoimmune diseases [44]. 

Lastly, toll-like receptors (TLR) are an adjuvant mainly used for nucleic acid and protein-based COVID-19 vaccines, including TLR4 and TLR7 [45]. The adjuvant can induce the production of Interleukin-1, which can activate alveolar macrophages. Moreover, it helps neutralize antibodies and CD4+ T cells, resulting in optimal protection against COVID-19 through the synergistic effect of CD8+ T cells [46]. The side effects can be mild or moderate, including flu-like symptoms [47].

Additionally, the route of administration and the formulation are crucial to the immunization outcome [37]. All COVID-19-approved vaccines are injected through the intramuscular route, as it shows an efficient immune response. Although subcutaneous injection can be effective, it may also cause severe side effects at the site of injection [48]. An under-investigation newly developed route of the COVID-19 vaccine is inhalation. However, immunization through inhalation on rats showed high IgG and IgA antibodies, which may be the future of vaccination against airborne pathogens [37,49]. 

Storage and transportation are also essential for COVID-19 vaccines. Table 2 summarizes the transportation and storage of WHO-approved COVID-19 vaccines. In developing countries, complications related to transportation and storage were raised because of the need for freezing temperatures, especially for mRNA vaccines, to maintain vaccine stability during transport [50]. Therefore, many studies were conducted to enhance the strength and supply of COVID-19 vaccines; one of these methods is using lyophilized COVID-19 vaccines, which are being tested by a Korean drug company [51]. A scientist also claimed that a liposome-based liquid vaccine was successfully developed and could be potentially used for the SARS-CoV-2 virus [52]. This method may be globally beneficial in the future in case of any outbreaks and might make transporting and storing vaccines easier. Collectively, it is necessary to design, store, and transport vaccines within their approved specifications to have an optimum vaccine that involves fewer complications and better responses.

**Table 2:** Storage and transport of COVID-19 vaccines.

**Vaccine**	**Storage and Transport**	**References**
Pfizer/BioNTech (12 + formulation)	The vials can be stored between −90 °C and −60 °C until the expiration date and shipped thermally using dry ice as they are stable for 30 days. They can be kept in the freezer for up to 2 weeks and in the refrigerator for up to 1 month (31 days).	[53]
Moderna	This vaccine should be stored at −20 °C. It is stable for around 1 month between 2 and 8 °C Unpunctured vials can be stored in a refrigerator from 2 to 8 °C for up to 30 days. Punctured vials can be held between 8 and 25 °C for 24 h.	[54]
AstraZeneca	This vaccine is stored, carried, and handled at normal refrigerated conditions between 2 and 8 °C for at least 6 months.	[55]
Sinopharm	The vials should be stored at a normal fridge temperature from 2 to 8 °C.	[54]
Sinovac	The vials should be stored at a normal fridge temperature from 2 to 8 °C for 12 months, and at room temperature not to exceed +25.	[54]
Covaxin	The vials should be stored at a normal fridge temperature from 2 to 8 °C for 6 months.	[54]
Convidecia	The vials should be stored at a normal fridge temperature from 2 to 8 °C for 12 months.	[56]
Johnson and Johnson	Opened vials should be discarded after 6 h. The vials should be stored at a normal fridge temperature between 2 and 8 °C for 3 months.	[54]
Covovax (Novavax)	The vials should be stored between 2 and 8 °C until the expiration date. They should be discarded 6 h after puncture.	[57]

## 3. Effectiveness of SARS-CoV-2 Vaccines

### 3.1. Pfizer/BioNTech

A lipid nanoparticle-based BNT162b2 vaccine encoding the full-length SARS-CoV-2 S protein is modified by two proline mutations to lock it in a prefusion form [26]. The efficacy trial included 43,548 participants who were randomized to receive the BNT162b2 vaccine or a placebo [26]. They concluded that in 16 years old and above, two doses of BNT162b2 inferred 95% protection against COVID-19 [26]. However, according to another pivotal study, four months after the second dose, efficacy declined by an average of 6% every two months, from 96.2% to 83.7% [58]. In addition, a new study suggests that two doses of the Pfizer-BioNTech vaccine were less effective against a more contagious strain. The Pfizer-BioNTech vaccine was 93.7% effective against the Alpha variant and 88.0% effective against the Delta variant when given in two doses [59]. The vaccine’s effectiveness against the Omicron variant was 65.5% from 2 to 4 weeks, dropping to 8.8% at ≥25 weeks after two Pfizer doses. The booster increased the vaccine’s effectiveness to 73.9% from 2 to 4 weeks, then declined to 64.4% from 5 to 9 weeks [60].

### 3.2. Moderna 

Moderna vaccine is an mRNA-based vaccine encapsulated in a lipid nanoparticle that encodes a full-length spike protein of the SARS-CoV-2 in a prefusion-stabilized conformation [61]. A phase III randomized trial was conducted at 99 different centers across the US, in which participants were assigned to receive two doses of the mRNA-1273 vaccine or a placebo. A 94.1% efficacy was achieved by the Moderna vaccine [62]. A study was conducted on 26,683 COVID-19-positive patients, 16% (Delta infection) and 84% (Omicron infection), and over 67,000 who tested negative [63]. They concluded that in those who received two doses of the Moderna vaccine, the effectivity was 44% against the Omicron variant until three months after vaccination, with a decline in effectivity afterward. However, after three doses, increased effectiveness was noticed (94% against Delta and 72% against Omicron) within two months of receiving the vaccine. For immunocompromised patients, the effectiveness was only 29% [63].

### 3.3. AstraZeneca 

The viral vector vaccine, AstraZeneca, uses a harmless virus as a delivery mode to carry the genetic material of the surface spike protein of SARS-CoV-2. It allows the cells to activate the immune system when the body reencounters the disease [64]. The virus vector generally used is ChAdOx1, or adenovirus, a virus known to induce the common cold in chimpanzees. This virus is modified not to cause harm or infection to the human body [64]. Many trials were performed to study how effective the immune response of AstraZeneca is towards hospitalization and the reduction of the symptomatic effect of COVID-19. A phase III randomized trial in the US was conducted by comparing two doses of the AstraZeneca vaccine to the placebo group [65]. The vaccine showed 79% efficacy in preventing severe symptomatic events and 100% in reducing the number of infected patients admitted to the hospital. 

Another study was performed in Canada after the appearance of new, more contagious variants of SARS-CoV-2, such as Delta and Gamma, to evaluate how effective the vaccine is in lowering the risk and improving protection against both variants. Results from the Canadian Immunization Research Network against symptomatic events caused by different variants showed that after one dose of the AstraZeneca vaccine, the efficacy was reduced to 70% and 72% for Delta and Alpha variants, respectively [66]. At the same time, it was decreased to 50% for Beta and Gamma variants. In addition, for hospitalization, the efficacy was reduced to 92% and 86% for Delta and Alpha variants, respectively [66].

### 3.4. Johnson & Johnson 

Johnson & Johnson uses the same vaccine platform as AstraZeneca; the adenovirus is used as a vehicle to transport the genetic code of SARS-CoV-2 [67]. In clinical trials, a single dose of Johnson showed 66.3% effectiveness in preventing SARS-CoV-2 infection and 50% efficacy against symptomatic events [67,68]. Many studies suggested the need for Johnson & Johnson’s booster dose to strengthen the vaccine’s benefits. A recent study in South Africa demonstrated that a booster dose from Johnson & Johnson showed up to 85% effectiveness in reducing hospitalization [69]. Another study in the US showed that antibody titers increased four to six times after booster doses compared to a single dose of the vaccine [70].

### 3.5. Convidecia

Convidecia is a viral vector vaccine similar to those of AstraZeneca and Johnson & Johnson [71]. It is produced by genetic engineering using recombinant virus technology. The vaccine showed low efficacy in reducing the symptomatic events of COVID-19 in clinical trials compared to other vaccines. Its effectiveness ranged from 57.5 to 63.7%. The vaccine’s efficacy against severe COVID-19 ranged from 91.7% to 96% [71,72,73]. In the phase IV clinical trial, the company that developed the vaccine, CanSinoBio, suggested that a heterologous booster would give a better immune response as it would stimulate the body to produce more antibodies to fight the virus. The heterologous booster showed a six-fold increase in neutralizing antibodies compared to a homologous booster dose [74]. Another study was performed using a protein subunit vaccine (ZF2001) as a heterologous booster compared to a homologous booster of Convidicea that showed from a 2.5- to 3.3-fold increase in humoral immune response and was safer and more tolerable [75].

### 3.6. Sinovac-CoronaVac 

Sinovac vaccine follows the usual platform used for vaccination using an inactivated virus. The WHO follow-up on the immunogenicity of Sinovac in human clinical trials showed that adults aged 18 and above could be immunized using Sinovac in two doses [76]. However, the antibodies formed from the two doses usually decline after three months [76]. Moreover, a study in Chile on the age group of 60 years and older showed that the vaccine was 66.6%, 85.3%, and 89.2% effective against COVID-19 infection, hospitalization, and ICU admission, respectively [77]. For adults between 18 and 59 years old, the efficacy in preventing COVID-19 infection was 65.03% [78]. Another study showed that antibodies formed after two doses of Sinovac were significantly lower in older patients, with an average of 85.3% efficacy compared to adults with an efficacy of 97.4% [79].

### 3.7. Sinopharm 

Like Sinovac, Sinopharm is also an inactivated vaccine [80]. In 2021, WHO approved the Sinopharm vaccine for emergency use based on interim phase III clinical trial data since it prevents symptomatic diseases in 79% of cases in adults younger than 60 years old [81]. Additionally, participants in the phase III clinical trial were randomized to receive 1 of 2 inactivated vaccines developed from SARS-CoV-2 WIV04 and HB02 strains or a placebo. During an average follow-up duration of 77 days for WIV04, the vaccine efficacy was 72.8%, and for HB02, it was 78.1%. They reported only two severe cases in the placebo group [82]. A case-control study of vaccinated elderly individuals showed that 14 days after the second dose, the Sinopharm vaccine reduced the risk of symptomatic COVID-19 infection, hospitalizations, and mortality by 94.3%, 60.5%, and 98.6%, respectively [83].

### 3.8. Covaxin 

The last WHO-approved inactivated vaccine for emergency use was Covaxin. During the phase III trial in India, Covaxin showed 81% efficacy against SARS-CoV-2 infection [84]. However, another study was performed to determine the effectiveness of Covaxin against the Delta variant. The efficacy of the vaccine was 64% after two doses and 44% after one dose, respectively [85]. This can conclude that Covaxin is a moderately effective vaccine, but it might not fully protect individuals against the new mutant strains. Additionally, a study showed that after six months of the first and second doses of Covaxin, the seropositivity was reduced significantly in individuals aged 60 years and older compared to younger individuals. Furthermore, the vaccine-induced antibody titers, regardless of age, were decreased after 6 months of vaccination by 56% [86].

### 3.9. Covovax (Novovax)

Covovax is the only protein subunit vaccine approved for emergency use in COVID-19. In India, the Covovax vaccine was given to individuals who were 18 years old and above, but recently it has been approved to be given to children from 12 to 17 years old [87]. In the phase III trial in India, the vaccine showed 89.3% effectiveness against COVID-19 infection [88]. In addition, a study in the UK comparing the Covovax vaccine with a placebo revealed that the vaccine was 86.3% and 96.4% effective against Alpha and non-Alpha variants, respectively, with no reported hospitalizations or deaths after immunization [29]. Briefly, all WHO-approved COVID-19 vaccines reduced symptoms of infection and prevented complications associated with them.

## 4. COVID-19 Vaccines Booster Dose

Vaccinations worldwide have provided a high level of protection against COVID-19, but the pandemic has not ended despite the rapid development of vaccines. Therefore, a third booster dose of the vaccine was recommended [89]. Maximizing the vaccine’s neutralizing antibody formation is the key to better immunity and protection against the virus and its different variants. Various studies evaluated whether a booster dose was required or not. According to a study performed by the National Institute of Allergy and Infectious Diseases (NIAID) on the immunity following mRNA vaccination in 34 adults, participants showed a decline in the antibody titers formed by the vaccine after 199 days of the first dose. However, three months after receiving the second dose, antibodies formed remained elevated for an extended period [89,90]. Another study at Alexandra General Hospital in December 2020 evaluated how the body handles the neutralizing antibodies formed from mRNA vaccines over 2 weeks, 1 month, 3 months, 6 months, and 9 months in healthy participants [90]. The study results showed that after 9 months of being fully vaccinated, there was a significant decline in the level of neutralizing antibodies with a median inhibition of 66.23%, meaning 1/5 of the participants were not fully protected against SARS-CoV-2 infection. For participants with comorbidities, further investigations are needed since the effect of each comorbidity on the kinetics of vaccination is substantially varied [90]. Collectively, a booster dose is more effective in enhancing immunity in older individuals since the ability to produce antibodies is lower and the clearance of antibodies is faster than in younger individuals.

## 5. Adverse Effects Due to SARS-CoV-2 Vaccines

The incredible speed of developing SARS-CoV-2 vaccines led to skepticism and safety concerns among the public and healthcare providers. However, some common adverse effects, such as the natural body’s responses to the vaccine, were reported. They can range from minor reactions, including fever, pain, and rash, to severe side effects, including vomiting, diarrhea, and allergic reactions [91]. These common adverse effects are expected and observed during clinical trials. However, rare side effects, including thrombocytopenia, pneumonia, hepatic injury, myocarditis, and respiratory failure, were also reported [92,93], which may be developed in the long run and usually emerge when large populations are vaccinated. 

Many studies have been conducted to report possible side effects and evaluate the safety of the COVID-19 vaccination. Pfizer/BioNTech and Moderna vaccines utilize mRNA for immunization [94]. Since this is considered a new strategy, assessing its success through studying the adverse events and symptoms is essential. Therefore, a cohort study was performed in the United States (US) during a phase III trial by the Mayo Clinic and the Hospital Institutional Review Board for vaccinated people. A total of 52 million doses of the two vaccines (Pfizer/BioNTech and Moderna) were administered. Both vaccines showed high efficacy with partial safety [95]. Common adverse effects varied among individuals, including fever, chills, nausea, vomiting, myalgia, headache, fatigue, and swelling at the injection site. Seven days after the first vaccination, 19.6% of participants reported at least one adverse effect using the online survey. On the other hand, 18.3% of the placebo group reported side effects. Additionally, 14.7% of vaccinated participants and 15.8% of the placebo group reported at least one side effect after the second dose. There was no significant difference between the two groups using the two different vaccines [95], indicating that the mRNA COVID-19 vaccines are well-tolerated. Participants aged from 18 to 59 were randomized to receive Johnson & Johnson’s single-dose vaccine with reported local side effects. These local side effects include pain at the injection site, swelling, erythema, and any local reaction [96]. On the other hand, many non-serious systemic effects were also reported by vaccinated participants, such as headache, fatigue, myalgia, nausea, and fever, along with different systemic reactions. Headache and fatigue were the highest reported side effects, with 38.9% and 38.2%, respectively [96]. In addition, AstraZeneca participants reported mild to moderate adverse events similar to those reported by Johnson & Johnson during phase I and II trials, such as chills, muscle aches, and headaches [97]. In Europe, during phase I and II clinical trials, AstraZeneca and Johnson & Johnson vaccinated individuals reported blood clotting events. However, clinical trials were resumed later, as the reported cases were rare compared to the number of vaccinated individuals [98,99,100]. Another study showed that some participants experienced severe side effects such as kidney pain, blue lips and nails, enlarged lymph nodes, and irregular heartbeats [101]. 

Convidecia vaccine is the only vaccine administered using the following two methods: inhalation or intramuscular injection [100]. Comparing the side effects of Convidecia immunized participants to a placebo, 74% of vaccinated individuals reported at least one side effect. Common adverse effects were pain, headache, fever, and fatigue. All side effects were mild but significantly higher in the vaccinated group [102]. Nevertheless, no serious events were reported, which concludes that the Convidecia vaccine has good safety compared to other viral vector vaccines. The third and most utilized vaccination platform uses a whole inactivated virus to obtain robust humoral immunity. Two inactivated vaccines, the Sinopharm and Sinovac vaccines, were approved for emergency use by the WHO [103]. Trials for the Sinopharm vaccine included a wide variety of patients, ranging from children to the elderly [104]. During phases I and II clinical trials, side effects were mild to moderate and more predominant after receiving the first dose of Sinopharm compared to the second and booster doses. The most common side effects were pain and fever [104]. In a Chinese study, the use of Sinovac reported 15.6% side effects after the first dose and 14.6% after the second dose compared to the placebo [105]. The data after the first and second doses of vaccination are considered similar. The most common side effect was pain at the injection site, while the common systemic side effects were fatigue and headache [105]. A study conducted in Indonesia showed similar side effects following both doses of Sinovac; around 14%, 80%, 71–78%, and 4% reported fever, pain at the injection site, upper arm pain, and cough, respectively [106]. Both studies showed that Sinovac is well-tolerated in ages eighteen to sixty [106].

Covaxin is the first Indian whole inactivated virus vaccine designed for the COVID-19 pandemic [107]. A study performed in India to monitor adverse events after immunization showed that during the phase I clinical trial, 5% of the individuals reported side effects such as pain at the injection site, redness, and swelling, and 14% reported systemic side effects, including headache, fever, and fatigue. In addition, children had more side effects than older participants [108], concluding that the vaccine is safer for fifteen-year-olds.

An observational survey showed that 77.27% and 72.72% of the participants reported side effects after the first and second doses of Covaxin vaccination, respectively, with fever as the predominant adverse event after vaccination (AEFI) [109]. Lastly, the only protein subunit approved vaccine by the WHO is Covovax; two doses are recommended for 18 years and older [110]. During the phase II clinical trial in India, 40.7% of the vaccinated participants reported 96 adverse events. In contrast, 18% of the placebo group reported 11 adverse events [111], and most side effects were mild and resolved without intervention. Overall, the approved COVID-19 vaccines were well tolerated, with only mild to moderate adverse reactions.

## 6. Vaccine Immune Responses

### 6.1. Beneficial Responses in Healthy Individuals

According to a St. Jude Children’s Research Hospital report, herd immunity is not superior to vaccination since both produce similar T cell responses [112]. The following two types of immune cells are activated: the B cell, which produces antibodies to combat the virus, and the T cell, which destroys the infected cells. Once the immune system responds to the vaccine, the level of antibodies decreases, but some B and T cells remain in the body to fight subsequent infections [113]. Kim et al. reported that all participants (43 healthy people who were administered two doses of the Pfizer-BioNTech vaccine) developed memory B cells against the spike protein of SARS-CoV-2 for six months following vaccination. In addition, the vaccine was also associated with antigen-binding IgG and neutralizing antibodies [21,114].

Adenovirus vector and mRNA vaccines stimulate the immune response by producing anti-S and anti-receptor binding domain (RBD) antibodies. They cause early IgA, IgM, and IgG antibody production and a long-lasting memory of B and T cells [16]. The neutralization antibodies were evident in all participants in the phase I trial after the second dose of the Moderna vaccine [61]. In phase I–IIa trials, 29 days after the first dose of the Johnson & Johnson vaccine, 90% of participants developed neutralizing antibodies to the wild-type virus. In participants between 18 and 55 years old, CD4+ T cell responses were detected in 76–83% of the cases [115].

**Figure 6:**
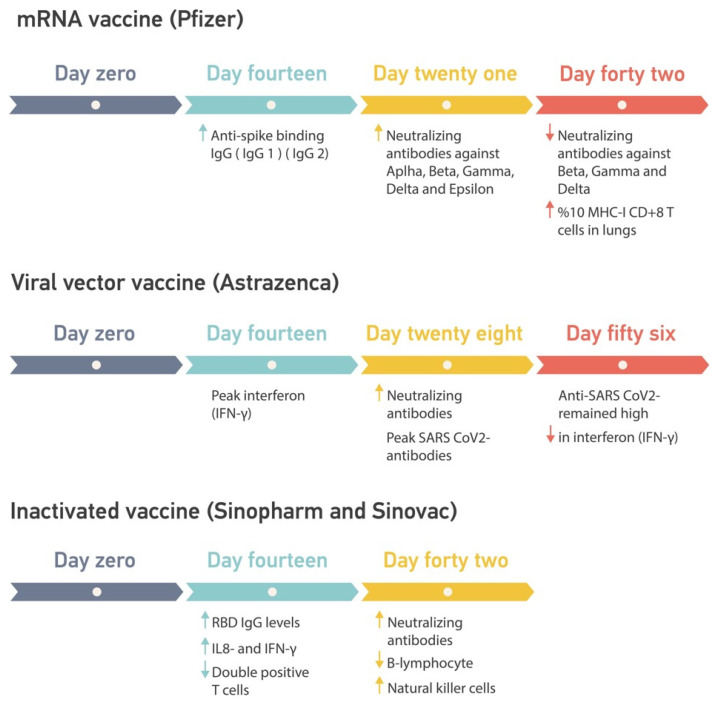
A time-course graph for immunological response in the different vaccination procedures.

Sinopharm/BBIBP-CorV vaccine recipients were tested for SARS-CoV-2 antibodies 4 weeks, 2 weeks, and 12 weeks after the first dose. Antibody responses declined insignificantly between 6 and 16 weeks. In addition, for the S1 peptide pool (peptides 1–130) and the S2 peptide pool (peptides 131–253), T and memory B cells showed positive responses in the ex vivo testing. However, the antibodies blocking ACE-2 receptors decreased significantly in all age groups [116]. An immunological response to various vaccination procedures is visualized on a time-course graph (Figure 6).

During a recent study, five adjuvant groups showed significant increases in CD4 cells after the second dose of a subunit vaccine. CD4 cells have two subsets, TH1 and TH2; TH1 is a CD4 responsible for the cytokines, such as TNF, associated with inflammation, while TH2 secretes cytokines, such as IL-4, associated with humoral immunity [117]. TH1 showed a slightly higher percentage of cells due to the CpG-alum adjuvant, while alum showed a higher TH2 rate. Aside from the fact that adjuvants enhance immunity, different adjuvants can elicit antibodies that can neutralize various antigens [117]. Therefore, the adjuvanted RBD-NP vaccine promotes protective immunity against SARS-CoV-2.

The third dose of mRNA vaccines (Pfizer/BioNTech or Moderna) helps induce an immune response similar to hybrid immunity (immune protection in those who received one or more COVID-19 vaccine doses and were infected at least once with SARS-CoV-2) [118,119]. A 5.82-fold increase in neutralizing activity was observed in participants who received the three doses (SN3) compared to the two doses (SN2) [120]. In addition, an increase of 2.2-fold in the secretion of neutralizing antibodies by S-specific B-lymphocytes in SN3 compared to SN2 was observed. Furthermore, three doses enhanced the binding affinity to the S protein and its receptor-binding and N-terminal domains [120]. Table 3 and Table 4 illustrate the beneficial immune responses, including IgG anti-RBD levels in the mRNA and inactivated SARS-CoV-2 vaccines, respectively. 

The clinical trial evaluating a booster dose of the same or a different vaccine reported an increase in the neutralizing antibodies to the Omicron BA.1 sub-lineage [121]. Despite this, antibody levels were lower in those who initially received the adenovirus vector vaccine or as a booster. Three months after the boost, neutralizing antibody levels declined 2.4–5.3-fold across all groups. Comparatively to the BA.1 sub-lineage, sub-lines BA.2.12.1 and BA.4/BA.5 were 1.5 and 2.5 times more resistant to neutralization, respectively [121]. The trials and studies emphasize the efficacy of the vaccines in stimulating the immune system; however, the effect on different strains needs to be better studied.

Moreover, immune responses are associated with age, as Tazerji et al. reported that death in the elderly is associated with decreased function of the immune system [122]. Generally, the COVID-19 vaccine stimulates neutralizing antibodies and IgG, both of which have beneficial effects on the immune system.

**Table 3:** Beneficial immune responses in mRNA COVID-19 vaccines.

IgG Anti-RBD Levels BAU/mL			
mRNA Vaccines			
Data	Pfizer	Moderna	References
Self et al.	2950	4274	[123]
Kanokudom et al., (Pfizer); Al-Sadeq (Moderna) et al.	2584	2272	[124,125]
Median	2767	3273	
Standard deviation	258.801082	1415.62778	
Fold change	1.14164087	1.88116197	

The result of study 2 in Moderna was in 1.6 × 104 AU/mL. Then, the value was multiplied by 0.142 to convert it to binding antibody units per milliliter (BAU/mL), which is 2272 (BAU/mL).

**Table 4:** Beneficial immune responses in inactivated COVID-19 vaccine.

Sinopharm Vaccine			
Data	IgG Levels BAU/mL	Number of Participants	References
Alqassieh et al.	170 (60–400)	*n* = 147	[126]
Kanokudom et al.	164.1 (133.8–201.1)	*n* = 60	[125]
Median	167.05	103.5	
SD	4.171930009		
Fold	1.035953687		

### 6.2. Harmful Responses in Healthy Individuals

Vaccination has led to a few severe events, including vaccine hypersensitivity reactions reported on different vaccine platforms. The leading cause of hypersensitivity can be associated with excipients such as stabilizing agents, preservatives, or adjuvants, and to a lesser extent, due to the active antigen used [127,128]. It was observed that PEG surfactant was the cause of allergy in the mRNA vaccines from Pfizer/BioNTech and Moderna; individuals with skin disorders can be susceptible to this type of adverse reaction. It is believed that EDTA, a preservative used in viral vector vaccines such as AstraZeneca, is responsible for causing sensitivity in these vaccines [128]. The vaccine’s active antigen can be another source of hypersensitivity since the human body might consider the functional antigen as a foreign body and elicit an allergic reaction [128]. Moreover, COVID-19 vaccination has also been shown to cause the reactivation of Herpes Zoster through stimulation of toll-like receptors (TLR), production of interferons, and activation of immune cells, which can lead to the reactivation of latent viruses [129]. Several factors contribute to herpes zoster (HZ) development, including age-related declines in cell-mediated immune responses to the Varicella zoster virus (VZV). VZV reactivation is caused by the T cell compartment’s failure to keep the virus under control; this is anticipated to happen more frequently as people age due to the immune system’s age-related dysfunction and decline (immunosenescence) [130]. Seven HZ cases have been reported following an mRNA vaccine (five cases reported and six observed), indicating a temporal link between COVID-19 vaccination and the development of HZ [131]. However, there is no specific mechanism for developing HZ after infection or vaccination with COVID-19 In AstraZeneca, the adenovirus used as a vector binds strongly to platelet factor 4 (PF4) and activates platelets [132]. In addition, a previous study found a positive correlation between serum reactivity and PF4 in vaccine-induced immune thrombotic thrombocytopenia patients with COVID-19, suggesting a possible causal relationship [133]. Harmful immune reactions were few and mainly caused by hypersensitivity to vaccine formulations.

### 6.3. Beneficial Responses in Unhealthy Individuals

A significant association was found between steroid use and a lack of T cell responses [134]. Patients who receive specific medications and have certain solid tumors and hematologic malignancies could not sufficiently produce anti-SARS-CoV-2 IgG antibodies after immunization [135]. The COVID-19 mRNA vaccine generally elicited excellent humoral responses but insufficient cellular responses in patients with solid cancer (Table 5), while hematologic malignancies elicited less appropriate humoral and cellular responses. Most cancer patients were unable to elicit a CD8+/CD4+ T cell response due to immunosuppressant medications [134]. A marked decrease in neutralizing capacity of 26.3% at 1 month and 43.6% at 3 months in hematologic malignancies patients were reported [136]. On observation, there was a uniform blunting of responses in patients with leukemia, lymphoma, and multiple myeloma. Some targeted anticancer therapies inhibit immune responses, but single-agent immunomodulating agents do not [136]. There are no differences in anti-receptor binding domain (RBD) IgG responses between HIV-positive individuals with CD4+ counts over 250 cells/mm^3^ and the general population. In contrast, CD4+ counts less than 250 cells/mm3 have a weaker response [137]. Further, other studies on HIV-positive vaccinated individuals showed increased immune responses compared to individuals with lower CD4+ counts. Taken together, HIV patients with CD4+ counts of ≥350 cells/µL have an S-RBD-IgG titer 2 folds higher than HIV patients with <350 cells/µL CD4+ counts. However, HIV patients with ≥500 cells/µL CD4+ counts have an S-RBD-IgG titers 1.25 folds higher than HIV patients with <500 cells/µL CD4+ counts (Table 6). Multiple sclerosis (MS) patients who received no treatments developed protective SARS-CoV-2 humoral responses similar to those of healthy individuals after COVID-19 vaccines. However, the immune response to COVID-19 vaccination varied between MS patients treated with high-efficacy disease-modifying therapies (DMTs) [138]. In another study, similar results of protective immune responses for the untreated MS patients and healthy individuals’ group were reported. For the fingolimod treatment, 9.5% developed humoral immune responses, compared to 3.8% from the previously discussed article findings. The low lymphocyte count in most MS patients who received fingolimod might have contributed to the failure to produce SARS-CoV-2 IgG antibodies [139]. Table 7 compares the two discussed studies. Most solid organ transplant recipients who received two doses of the mRNA SARS-CoV-2 vaccination had measurable antibody responses after the second dose. However, those who did not react to dose one often showed low antibody levels [140]. 

A study reported that after receiving two doses of the mRNA BNT162b2 vaccine (Pfizer-BioNTech), none of the lung transplant recipients (LTRs) produced anti-SARS-CoV-2 antibodies, whereas 85% did so upon SARS-CoV-2 infection. Testing specific CD4+ and CD8+ T cell levels in immunocompromised patients post-vaccination would benefit the assessment of immune responses. Additional boosting may be necessary for developing an antibody response in LTRs after vaccination [141]. To a large extent, immune responses were also evident in unhealthy individuals.

**Table 5:** Beneficial immune responses in solid tumor after taking mRNA vaccines.

Solid Tumor Cases				
Study	Number of Cases	Average	Standard Deviation	Median
Ehmsen [142]	139	152.5	19.091	152.5
Mencoboni [143]	166			
Solid tumor antibodies				
Study	Anti-spike SARS-CoV-2 IgG in BAU/mL after mRNA vaccine	Average	Standard deviation	Median
Ehmsen (three doses of mRNA vaccine)	2464	1787.65	956.503343	1787.65
Mencoboni (two doses of mRNA vaccine)	1111.3			

**Table 6:** Beneficial immune responses in HIV patients after taking COVID-19 vaccine.

Study	HIV Patients CD4+ Counts	Antibody Titer (S-RBD-IgG Titers) U/mL in Inactivated Vaccine	Average	Standard Deviation	Median
Liu et al. [144]	≥350 cells/µL	22.4	16.8	7.919595949	16.8
Liu et al. [144]	<350 cells/µL)	11.2			
Netto et al. [145]	≥500 cells/μL	53.3	47.95	7.566042559	47.95
Netto et al. [145]	<500 cells/μL	42.6			

**Table 7:** Number of vaccinated cases among multiple sclerosis patients.

Study 1 [138]		Study 2 [139]	
Multiple Sclerosis	Healthy Individuals	Multiple Sclerosis	Healthy Individuals
Untreated N = 32	*n* = 47	Untreated N = 76	*n* = 89
Cladribine N = 26		Cladribine N = 48	
Ocrelizumab N = 44		Ocrelizumab N = 114	
Fingolimod N = 26		Fingolimod N = 42	

### 6.4. Harmful Immune Response in Unhealthy Individuals 

A previous study reported a few patients with a flare-up onset of autoimmune rheumatic disease (AIRD) after the COVID-19 vaccine, which was due to the adjuvant use [146]. There has been speculation that vaccine adjuvants could trigger different autoimmune reactions by stimulating inflammatory products, which cause bystander T cells to become activated; however, the exact mechanism remains unclear. In addition, there needs to be more detailed information about systemic reactions following vaccinations [147]. For example, Campos et al. observed a relapse of autoimmune cytopenia after SARS-CoV-2 vaccination [148]. They claimed it happened through the stimulation of preexisting B cells to produce autoantibodies and by molecular mimicry (a molecular structure that affects the construction of another molecule). Therefore, blood monitoring and a high dose of dexamethasone are required for those patients to prevent unwanted immune responses.

Following the COVID-19 vaccination, several cancer patients have been detected to have metabolically active axillary lymph nodes. For example, in a 62-year-old man diagnosed with prostate cancer, imaging showed that his left axillary, paratracheal, para-aortic, subcarinal, and hilar lymph nodes were enlarged 3 weeks after vaccination [149]. Immunization caused local inflammation at the injection site, affecting the lymph nodes afferent to the injection site and the muscles around the injection site [149]. 

The development of glomerulonephritis (GN) after the mRNA vaccine results in a more potent immune response [150]. This might be due to the upregulation of CD4+ and CD8+ T cells as part of the cell-mediated response and an increase in interferon γ secretion. In situations where immunogenicity and cross-reactivity are higher, immune activation is likely to occur unexpectedly [150], unmasking or instigating autoimmune processes that may aggravate, disguise, or exacerbate existing conditions [151]. Of the 13 patients studied who showed GN, 62% were newly diagnosed, whereas 38% relapsed. It was observed that five patients developed symptoms after the second dose, and three patients developed symptoms after the first dose [150]. Patients with confirmed systemic lupus erythematosus (SLE) flares following vaccination required a change in the treatment in 71% of cases and hospitalization in 19% of cases [152]. Autoimmune responses may be triggered by the interaction between mRNA vaccines and cytoplasmic RNA-binding proteins involved in the post-translational regulation of inflammatory mediators [152]. Principally, most of the harmful immune responses in unhealthy individuals were a flare-up of a pre-existing condition.

## 7. Future Perspective 

To combat the recent COVID-19 pandemic, companies have demonstrated several technologies to advance the vaccine development. For instance, nanotechnology has dramatically accelerated the development of vaccines, enabling a nano-level investigation that allows the researchers to better imitate the interaction between the virus and the immune system [153]. The nano vaccines protect the antigen from biodegradation through the depot effect, providing slow release. Nanotechnology also improves antigen drainage and accumulations in lymph nodes due to their small size. They also enhance the uptake by antigen-presenting cells. Lipids are known as an excellent transporter of nucleic acids to the cells, mainly due to their compatibility with the lipid cell membranes [154]. Although mRNA-based vaccines have never been approved, the nucleoside-modified mRNA-lipid nanoparticles (LNP) vaccine platform employed by Pfizer/BioNTech and Moderna in developing SARS-CoV-2 vaccines has extensively undergone preclinical studies for its effectiveness and supportive protective effect of the humoral immune responses [155]. Furthermore, angiotensin-converting enzyme-2 (ACE-2) receptors are prime targets for antibody-mediated inactivation of SARS-CoV-2 by preventing its binding to RBDs, limiting its propagation, and spreading in the host [156]. For targeting pathways, various epitopes such as S, M, N, and E proteins have been tested for their ability to enhance antibody production and T cell responses against SARS-CoV-2 [157]. Vaccines targeting the receptor binding motif in the S1 subunit of the S protein may not be effective because of the significant genetic mismatches in structures targeting this region, while linear epitopes of the S2 subunit may induce a protective antibody response [158]. On the other hand, targeting the RBD prevented the virus infection by preventing entry into the host cells [158,159].

## 8. Conclusions 

An unanticipated, highly contagious respiratory pathogen was suddenly discovered as the cause of the emergence of the COVID-19 pandemic, necessitating the rapid development and testing of new vaccine platforms. This review sheds light on the WHO-authorized emergency use vaccines, including Pfizer/BioNTech, Moderna, Sinopharm, Sinovac, Covaxin, AstraZeneca, Convidecia, Johnson & Johnson, and Covovax. Additionally, we have discussed the effectiveness and protection of different vaccines, their safety concerns, the common side effects that individuals may encounter, the antibodies’ formation, and the beneficial and harmful immune responses. As a result, several vaccines utilizing viral vectors, nucleic acids, protein subunits, and inactivated viruses have been developed and become available on the market. However, many others are still under clinical trials for further investigation.

## Data Availability

Not applicable.
